# Understanding the dynamics in distribution of invasive alien plant species under predicted climate change in Western Himalaya

**DOI:** 10.1371/journal.pone.0195752

**Published:** 2018-04-17

**Authors:** Sunil Thapa, Vishwas Chitale, Srijana Joshi Rijal, Neha Bisht, Bharat Babu Shrestha

**Affiliations:** 1 International Centre for Integrated Mountain Development (ICIMOD), Kathmandu, Nepal; 2 Tribhuvan University, Kathmandu, Nepal; Shandong University, CHINA

## Abstract

Invasive alien plant species (IAPS) can pose severe threats to biodiversity and stability of native ecosystems, therefore, predicting the distribution of the IAPS plays a crucial role in effective planning and management of ecosystems. In the present study, we use Maximum Entropy (MaxEnt) modelling approach to predict the potential of distribution of eleven IAPS under future climatic conditions under RCP 2.6 and RCP 8.5 in part of Kailash sacred landscape region in Western Himalaya. Based on the model predictions, distribution of most of these invasive plants is expected to expand under future climatic scenarios, which might pose a serious threat to the native ecosystems through competition for resources in the study area. Native scrublands and subtropical needle-leaved forests will be the most affected ecosystems by the expansion of these IAPS. The present study is first of its kind in the Kailash Sacred Landscape in the field of invasive plants and the predictions of potential distribution under future climatic conditions from our study could help decision makers in planning and managing these forest ecosystems effectively.

## Introduction

Biological invasion has become one of the major causes of economic and environmental damage in most of the countries across the world [1–3] and its impacts have been predicted to increase even further under future climatic conditions [4–5]. The Convention on Biological Diversity (1992) emphasized biological invasion as one of the major driver of biodiversity decline and the second biggest threat after habitat destruction and ecosystem degradation [6–9]. Climate Change, anthropogenic pressure, and land use change have accelerated bio-invasion [10–11].

The existing unique landscapes, ecosystems, and biota of Hindu Kush Himalayas (HKH) are spawned as a result of diverse climatic, topographic, geological, and altitudinal variations [12]. The HKH region is sensitive and fragile to climate change and is experiencing an annual increase in temperature from 0.03–0.07°C [13–14]. Plant invasions in mountain areas are likely to rise because of increase in trade, tourism activities, climate change, and anthropogenic disturbances and can alter both native floral and faunal species composition inducing prolonged negative impacts [8, 15–16]. Over the last few decades, the study of invasions has received much more attention globally and there are a lot of studies focused on mapping the distribution of invasive species, quantifying economic and ecological impacts, and developing efficient and economical management approaches [17–19]. However, the potential expansion of the invasive plants in the HKH region under predicted climate change remains underexplored, especially in the transboundary context that compels us to undertake this study. Our study provides baseline information for understanding the current distribution and predicting the future distribution of invasive alien plant species (IAPS) which are currently in the early stage of invasion and which could be potential threats in the future.

Limited environmental and historical bioclimatic data and systematic monitoring, coupled with political sensitivities within the region result in the lack of knowledge base, which is crucial for developing scientifically comprehensive transboundary climate change adaptation strategies. Therefore, there is an urgent need for regional cooperation in the HKH region for informed decision-making, risk and vulnerability mapping, effective biodiversity, and conservation management and holistic approach while developing the climate change adaptation strategies [14].

Species distribution models (SDMs) are scientifically proven tools for assessing and predicting the impacts of climate change on flora and fauna [20–23]. SDMs can be used to determine the relationships between species and their environment and predict their distribution from occurrence (presence only or presence/absence) data [24–27]. Understanding the factors influencing species distribution is imperative for ecological research in developing effective adaptation strategies to cope with impacts of climate change [28–29]. Selecting the most suitable modelling algorithm and relevant datasets is a major challenge in species distribution modelling [30]. SDMs dealing with presence-only data might be more advantageous over presence/absence modeling methods, conditional to the suitability for the study [20, 31] eg. MaxEnt.

Maximum entropy (MaxEnt) method is a general purpose machine learning method applied for producing species distribution maps using presence-only data [29, 31–32]. MaxEnt, a bioclimatic model is widely used by conservation practitioners and researchers as a tool for prediction and distribution of species [33]. Wilson et al., [34] used the combination of MaxEnt and Generalized Linear Mixed Models (GLMMs) to identify areas of high conservation value for the endangered species *Margaritifera margaritifera* (L.). Chitale et al., [20] used MaxEnt to predict the distribution of 637 endemic plants in four global biodiversity hotspots including the Himalaya, by combining climatic and non-climatic variables. Whereas Adhikari et al., [35] modelled hotspots of invasive plant species through Ecological Niche Modeling (ENM) using MaxEnt for guiding the formulation of an effective policy for controlling the IAPS. For instance, West et al., [36] used a MaxEnt model with an invasive species *Bromus tectorum* (cheatgrass) presence data and evaluated its usefulness in a management context. On the other hand, Qin et al., [37] investigated the spatial patterns of *Lantana camara* habitat changes from its current distribution to future potential occupied areas using the MaxEnt ecological niche modeling technique. In this study also, MaxEnt modelling was used as it can achieve high predictive accuracy [31]. As the sample size shrinkages the model accuracy decreased however, MaxEnt modelling is less sensitive than other approaches to the number of presence locations [31, 38]. MaxEnt tuned their regularization in relation to sample size to avoid over fitting [38–39]. Presence only data are good enough for species distribution modelling and the AUC scores obtained for predictions from it can be sufficiently accurate [39–40]. SDMs have some limitations such as overestimation of presence of species. Assumption of random sampling of presence of species on the grid cells made by SDMs predicts large probability of presence in each cell, which could be in fact overestimation [29]. MaxEnt uses presence only data and it may give high predicted values for environmental conditions outside the range [32]. In order to avoid the over estimation, we applied a threshold value 0.5 was applied in this study and used only those pixels that have values equal to or higher than 0.5.

In this paper, we use data of distribution of 11 invasive plants (namely *Ageratina adenophora* L., *Ageratum conyzoides* L., *Ageratum houstonianum* Mill., *Amaranthus spinosus* L., *Bidens pilosa* L., *Erigeron karvinskianus* DC., *Lantana camara* L., *Parthenium hysterophorus* L., *Senna occidentalis* (L.) Link., *Senna tora* L. Roxb. and *Xanthium strumarium* L.) distributed in four districts of the Kailash Sacred Transboundary Landscape viz., Darchula, Baitadi, and Bajhang in Nepal and Pithoragarh district in India. The species considered in this study have been reported as ‘invasive’ in national level assessments and mapping [41–42]. In Nepal and Himalayas, most of the invasive species have been spreading from southern lowland to mid hills and mountains in the north. Some species (e.g. *Ageratina adenophora*, *Lantana camara*) are already widespread in the study region while other species (e.g. *Parthenium hysterophorus*) are less abundant but they have already established small satellite population at a number of locations indicating their high potential to spread in the landscape. In absence of previous studies on invasive species in the region, it is not possible to estimate the duration for which the species is there. This could be an interesting topic of research for the future. The habitats known to be invaded by these species can be described as below:

Among them, *Ageratum conyzoides*, *A*. *houstonianum* and *Erigeron karvinskianus* are primarily invading agroecosystem; *A*. *adenophora* and *L*. *camara* in forest and shrublands; and the remaining six species in grazing lands, roadside vegetation and residential areas [43–44].

The objectives of the study were to i) determine the current distribution pattern and habitat of the selected invasive plant species; ii) use the occurrence data of the selected species to predict their change in distribution under simulated different climate change scenarios in 2050 and 2070; iii) support the formulation of guidelines and management practices on controlling and to provide information to prevent further spread of IAPS in the Kailash Sacred Landscape area based on the information obtained from modelling.

## Materials and methods

### Study area

**Fig 1:**
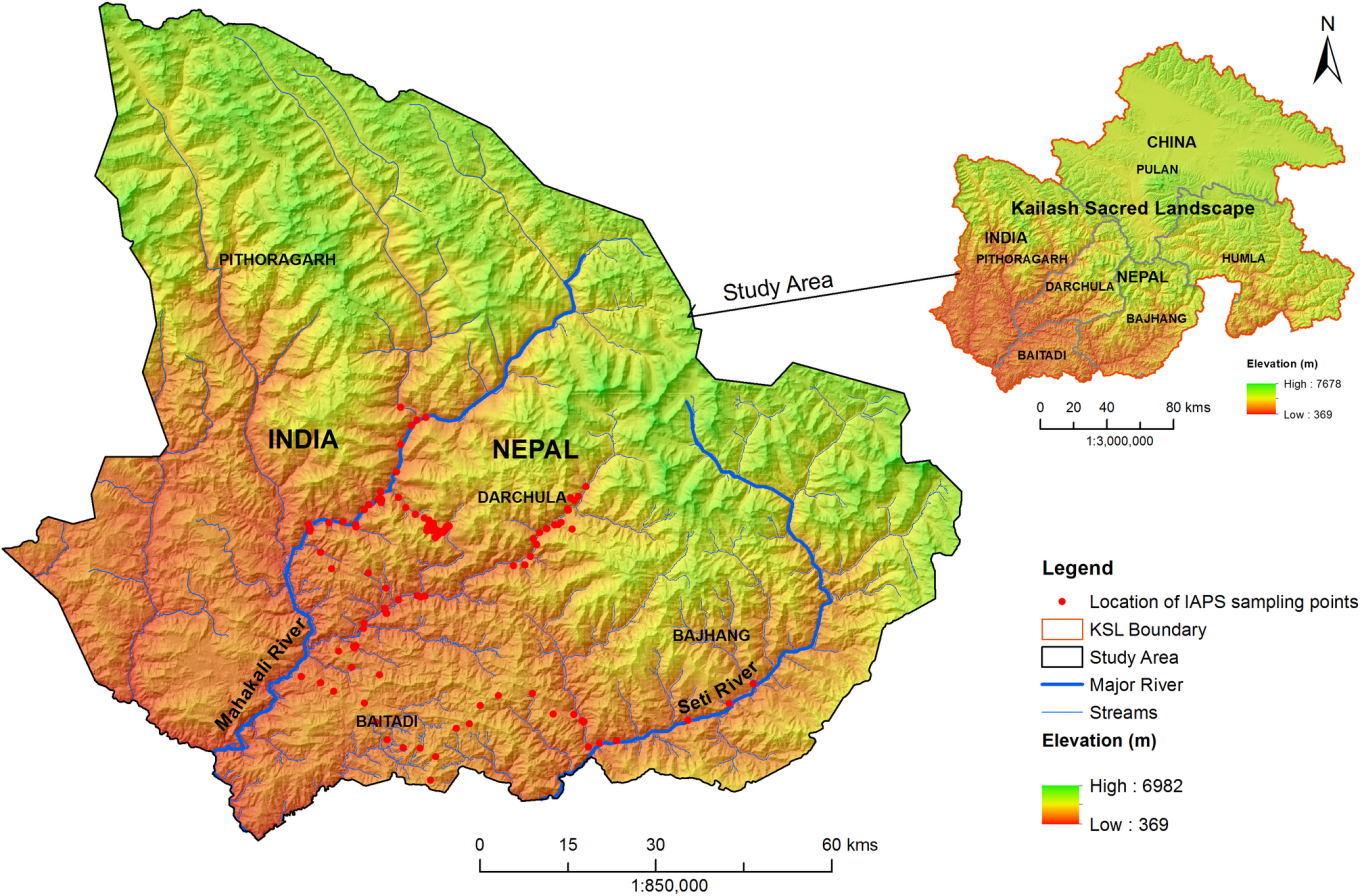
Location of IAPS field sampling points in study area.

The Kailash Sacred Landscape (KSL) spans across China, India, and Nepal. It represents a unique ‘transboundary cooperation’ and the first cooperation of its kind among China, India and Nepal [45–46], however, this study was focused only in certain regions of Nepal and India. The study area is located between 29.3° to 30.6° N latitudes and 79.86° to 81.56° E longitudes (Fig 1). The landscape covers an area of 13762 km^2^ and has a wide elevation range from 369 to 6982 m asl (above sea level). This geographic heterogeneity has given rise to a high level of biodiversity including an array of forest types ranging from moist subtropical broadleaf to temperate oak forests, alpine conifers, and pastures [47]. KSL is considered among the most revered sacred landscapes in the world and also the source of four of Asia’s most important rivers, the Brahmaputra, Indus, Karnali, and Sutlej [45–46].

### Data

#### Distribution of invasive alien plants

The current distribution of 11 invasive alien plant species (IAPS) in the study area was sampled using geographical positioning system (GPS) during 18–24 June 2015. Since the distribution of the IAPS was most dominant along the road, we recorded the coordinates of the locations of the species using Garmin GPS (GPSmap 62sc) along the road. Road networks often serve as conduit for dispersal of IAPS due to long distance dispersal of propagules by vehicles [48] as well as the road verges being suitable for colonization by the alien plant species [49–50]. Therefore roadside survey is used for rapid assessment of the diversity and distribution of the IAPS at landscape level [51–53]. In landscape level sampling, other methods of mapping is highly expensive and much more time consuming. Instead of single highway, we used several network of roads and trails for mapping. Furthermore, roadside environment provided suitable microhabitat for invasive plants which are ruderal in nature. Dispersal of all the invasive species considered in the study are dispersed directly or indirectly by human activities. However, *L*. *camara* seeds is additionally dispersed by birds too.

Interval between successive plots is subjective. Previous study in East Africa Wabuyele et al. used 25 km interval for the survey [51] while another study used 5–30 km interval [53]. We used 5–10 km interval for the survey. When elevation changes sharply, climate as well as the turn-over of species and vegetation also change shortly. The shorter interval in steep landscape was to capture all vegetation types adequately. It is not only the elevation, but in addition aspect and other topographic factors also change drastically in hill and mountains within a short distance. Therefore, shorter interval is required to adequately represent microhabitats.

From the starting point of the survey, at every 10 km distance, field plots of 10 m × 10 m were examined on both sides of the road to record distribution of IAPS. A section of road running south-north parallel to Nepal-India border in Dharcula (India) between Ghatibagar (south) and Tawaghat (north) was examined for the present of invasive alien plant species (IAPS) in every five kilometer road distance. In between these pre-defined locations, some opportunistic observations were also made if we encountered species not recorded in the immediate previous plot. This strategy was used in order to capture maximum possible records and locations of the distribution of IAPS. Altogether 15 IAPS were found in the Nepal region of the KSL; 11 of them with adequate occurrence data have been used in this study (Table 1). Remaining four species (*Galinsoga quadriradiata Ruiz* & *Pav*., *Ipomoea carnea ssp*. *fistulosa (Mart*. *ex Choisy*) *D*.*F*. *Austin*, *Oxalis latifolia Kunth*. *and Pistia tratiotes L*.) were found only at a few locations and thus excluded in the present analysis. In total 189 plots were examined during the survey.

**Table 1:** Invasive alien plant species recorded in the Kailash Sacred Landscape, Nepal and included in the present analysis.

Sl. No.	Scientific Name of IAPS	Common Names	Local Name	Family	Native Range	Sample Number
1	*Ageratina adenophora* L.	Crofton weed	Kalo Banmara	Asteraceae	Mexico	130
2	*Ageratum conyzoides* L.	Billygoat weed	Raunne/Gandhe	Asteraceae	Central & South America	*69*
3	*Ageratum houstonianum* Mill.	Blue billygoat weed	Nilo Gandhe	Asteraceae	Mexico & Central America	35
4	*Amaranthus spinosus L*.	Spiny pigweed	Kande lude	Amaranthaceae	Tropical America	*19*
5	*Bidens pilosa* L.	Blackjack/Hairy Beggar-tick	Kalo kuro	Asteraceae	Tropical America	*101*
6	*Erigeron karvinskianus* DC.	Mexican fleabane	Phule Jhar	Asteraceae	Mexico & Central America	*96*
7	*Lantana camara* L.	Lantana	Kirne Kanda	Verbenaceae	Central & South America	*20*
8	*Parthenium hysterophorus* L.	Parthenium weed	Pati Jhar	Asteraceae	Southern USA to South America	*25*
9	*Senna occidentalis* (L.) Link.	Coffee senna	Panwar	Leguminosae	Mexico to South America	*15*
10	*Senna tora* (L.) Roxb.	Sickle pod senna	Tapre	Leguminosae	South America	*28*
11	*Xanthium strumarium* L.	Rough cockle-Bur	Bhende Kuro	Asteraceae	America	40

For 9 out of 11 species, upper elevation limit of current distribution in the study region is lower than those reported from other parts of Nepal [43–44]. Therefore, it is less likely that the current uppermost distribution of these species has reached to climatic limit. Since dispersal of most of these species occurs through human activities, their further spread also depends on increasing human and livestock movements and opening of roads. However, species specific information on dispersal pattern and adaptation to high elevation region is not available for these study species.

#### Environmental and bioclimatic data

Nineteen bioclimatic variables of present and future time period (year 2050 and 2070) with a spatial resolution of 1 km^2^ were downloaded from worldclim datasets (www.worldclim.com). Based on an average annual change in means [54] we used projections of Community Climate System Model (CCSM4) under Representative Concentration Pathways viz., RCP 2.6 and RCP 8.5 for the year 2050 and 2070 as adopted by the IPCC in its Fifth Assessment Report (AR5). RCP 2.6 represents the lowest Greenhouse Gas (GHG) concentration pathway, whereas RCP 8.5 represents the extreme GHG concentration pathway [55] (Table 2). These data are statistically downscaled from a Global Circulation Model (GCM) using WorldClim 1.4 as baseline 'present' climate. Elevation, slope, and aspect were derived from digital elevation data based on the Shuttle Radar Topographic Mission (SRTM) at 90m spatial resolution. Finally, all ancillary layers were resampled to 1 km^2^ spatial resolution to match with the spatial resolution of climate variables.

**Table 2:** AR5 global warming increase (°C) projections.

Scenario	Mean and likely range	
	2046–2065	2081–2100
RCP 2.6	1.0 (0.4 to 1.6)	1.0 (0.3 to 1.7)
RCP 4.5	1.4 (0.9 to 2.0)	1.8 (1.1 to 2.6)
RCP 6.0	1.3 (0.8 to 1.8)	2.2 (1.4 to 3.1)
RCP 8.5	2.0 (1.4 to 2.6)	3.7 (2.6 to 4.8)

Source: IPCC, AR5 2014

### Modeling approach

MaxEnt software (version 3.3.3 k) downloaded from (http://www.cs.princeton.edu/~schapire/maxent/) was used in this study for predicting the distribution of 11 IAPS. MaxEnt generates an estimate of the probability of presence of the species that varies from 0 to 1, i.e. from the lowest to the highest probability of distribution. Species presence data and derived all environmental and physiographic data were converted to ASCII file before running the MaxEnt model. Models were built for individual species to predict the distribution of the IAPS under projected climate change scenarios.

Prediction accuracy and validation of the models were assessed on the basis of Area Under the Receiving Operator Curve (AUC), sensitivity (correctly classified presences) and specificity (correctly classified absences) [20, 56–57]. These measures are estimated from 578 random splits of the field dataset into a calibration subset with 70% of the data and a validation subset with 30% of the data, which is used by the model to assess the statistical significance [20]. AUC values range from 0 to 1. Values between 0.2–0.5 were considered low, 0.5–0.7 moderate and >0.7 as high while validating the model results. The jackknife procedure also called ‘leave one out’ was followed to assess the importance of variables [13, 56]. Jackknife, an alternative approach for assessing variable importance which provides statistics on the significance of each variable in the model [57–59].

### Image classification, combination, and analysis

The outputs were imported to ArcGIS 10.4 and converted to a.tiff raster format for further analysis. The output maps of the distribution of IAPS generated by MaxEnt were classified into two classes viz., 0.00 to 0.50 and 0.50 to 1.0. We selected pixels with or more 0.5 value to consider areas that depict at least 50% probability of species occurrence. The value greater than 0.5 depicts areas with a highly suitable habitat, while values lower than 0.5 represents low suitability of habitat for invasive plant species. Change detection maps were generated by using difference function in ArcGIS (subtracting future distribution from present distribution) for all IAPS to understand the expansion/ reduction/ no change in the distribution range of these species. The difference maps were then reclassified into three classes where negative values depicted range expansion, positive values depicted range reduction, and zero values depicted no change, and respectively. We quantified the changes in the altitudinal range in the distribution of these species by using the Digital Elevation Model (DEM).

## Results

### Prediction accuracy

Overall accuracy was high (~0.90, which refers to 90% accuracy) for predictions under present and future time periods. Prediction accuracy of the model used for analyzing the distribution under present and future time period of 11 IAPS were between 0.942 and 0.997 with training data and between 0.824 and 0.987 with test data, respectively (Table 3, S1 Table). Highest AUC value 0.997 was obtained for *L*. *camara* for the year 2070 in RCP 2.6. From the jackknife analysis, three variables out of a total of 22 bioclimatic physiographic variables used as predictor variables showed a major role in predicting the distribution of the IAPS (Table 4, S1 Fig) viz., (i) minimum Temperature of Coldest Month (Bio 6), (ii) mean Temperature of Driest Quarter (Bio 9), and (iii) mean Diurnal Range (Bio 2).

**Table 3:** Prediction accuracy of invasive species distribution modeling.

Scenario	Present		Year 2050				Year 2070			
			RCP 2.6		RCP 8.5		RCP 2.6		RCP 8.5	
	Training	Test	Training	Test	Training	Test	Training	Test	Training	Test
Minimum AUC Value	0.942	0.829	0.949	0.827	0.942	0.83	0.942	0.825	0.944	0.824
Maximum AUC Value	0.995	0.978	0.995	0.987	0.995	0.981	0.997	0.984	0.993	0.984

**Table 4:** Overall relative importance of predictor variables for invasive species from Jackknife test.

Time Period		Most Significant Variable	Code	Jackknife AUC Value
Present		Mean Temperature of Driest Quarter	Bio 9	0.90
2050	RCP 2.6	Mean Diurnal Range (Mean of monthly (max temp—min temp)	Bio 2	0.95
	RCP 8.5	Minimum Temperature of Coldest Month	Bio 6	0.89
2070	RCP 2.6	Mean Temperature of Driest Quarter	Bio 9	0.90
	RCP 8.5	Minimum Temperature of Coldest Month	Bio 6	0.92

### Distribution of invasive alien plant species

**Fig 2:**
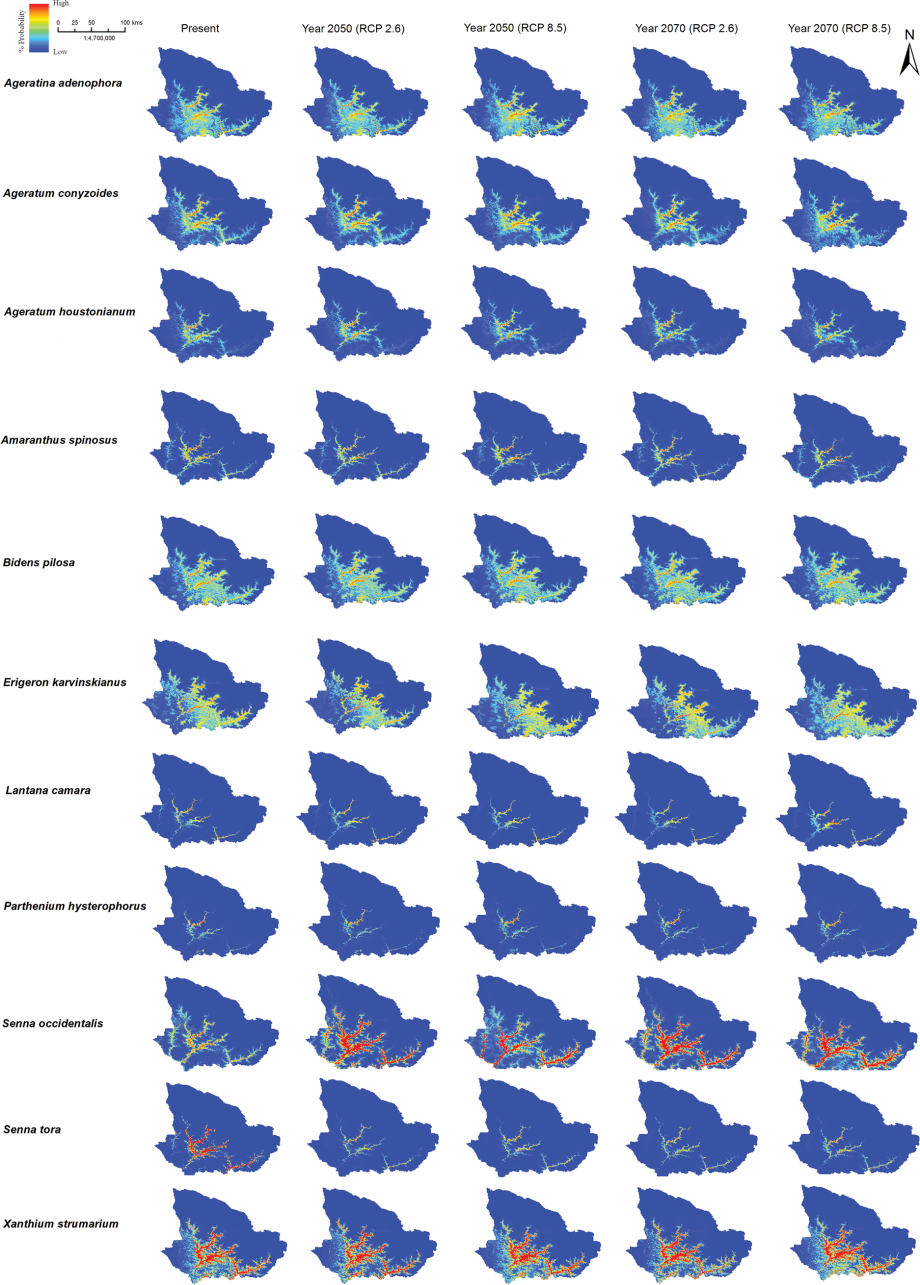
Predictive distribution of IAPS.

Under current climate conditions, the overall distribution of the IAPS is concentrated towards central South-East (SE) zone of the study area. However, the future distribution is predicted to shift towards North-East (NE) and expand to SE direction compared to the present distribution (Fig 2).

**Fig 3:**
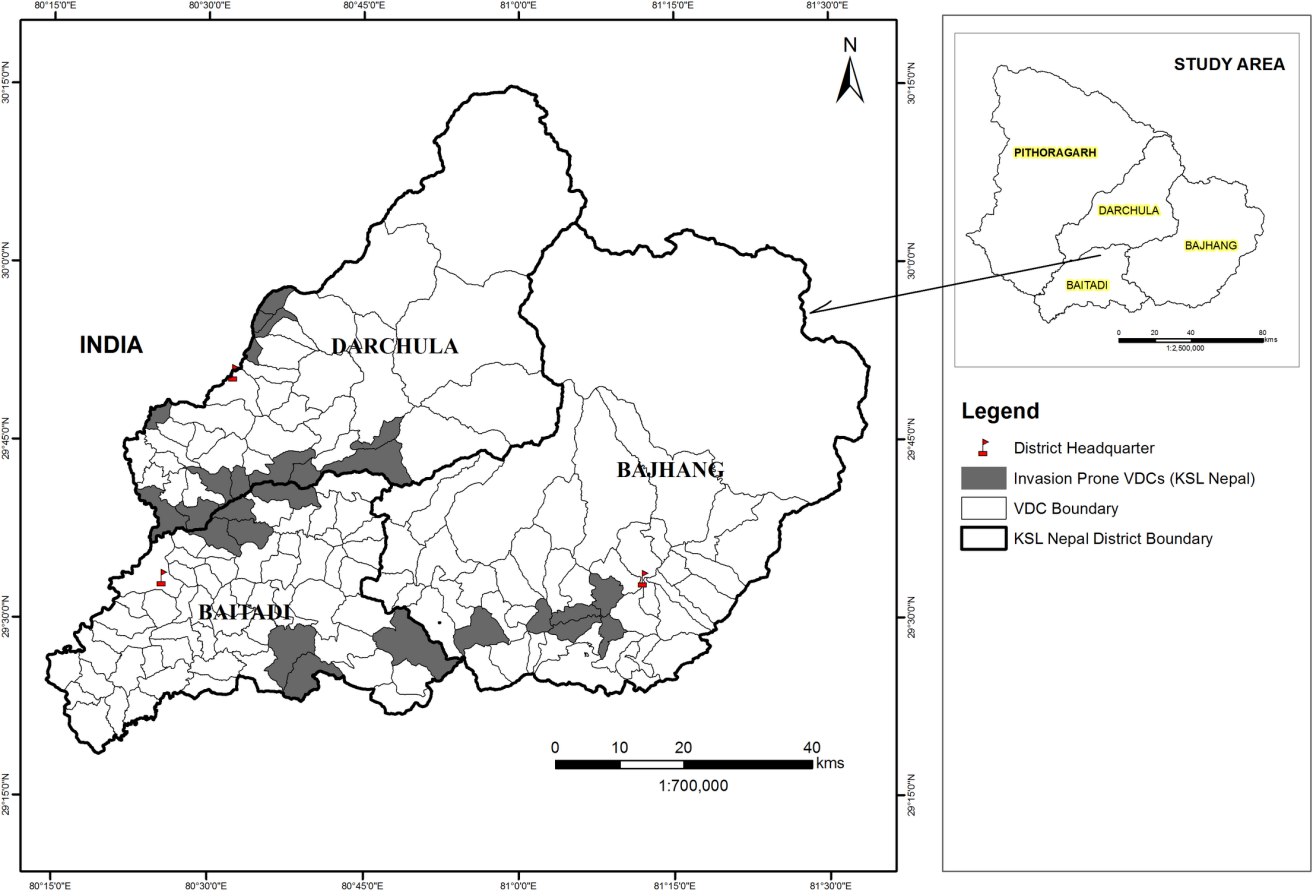
Highly susceptible areas of KSL Nepal to invasion by IAPS.

An overall expansion in the modelled distribution of a range of all 11 species is predicted under future climatic conditions under RCP 2.6 and RCP 8.5 by the year 2050 and 2070. Vegetation in the village development committee areas in the following districts of Nepal are highly susceptible to invasion by these IAPS: (i) Darchula district: Boharigaun, Bramhadev, Dattu, Dethala, Dhaulakot, Gokuleshwar, Huti, Lali, Pipalchaur, Sarmauli, Shikhar, Tapoban, (ii) Baitadi district: Gokuleshwor, Rim, Rudreswor, Shivalinga, Siddhapuri, Siddheswor, Sittad, (iii) Bajhang district: Chaudhari, Lamatola, Latinath, Malumela, Matela, Rayal, Subeda (Fig 3).

Even a moderate climate change scenario will lead to an expansion of the distribution of the 11 IAPS, which might pose threat to the native flora. Out of the 11 species, six are distributed in scrub vegetation and five in the subtropical needle-leaved forest. The current distribution of invasive plant species ranges between an altitude of 622 m to 2865 m; however, under future conditions (RCP 2.6 and RCP 8.5) the distribution is predicted to expand towards both upper and lower elevation ranging from 448 m to 3547 m.

### Range expansion in future distribution

**Fig 4:**
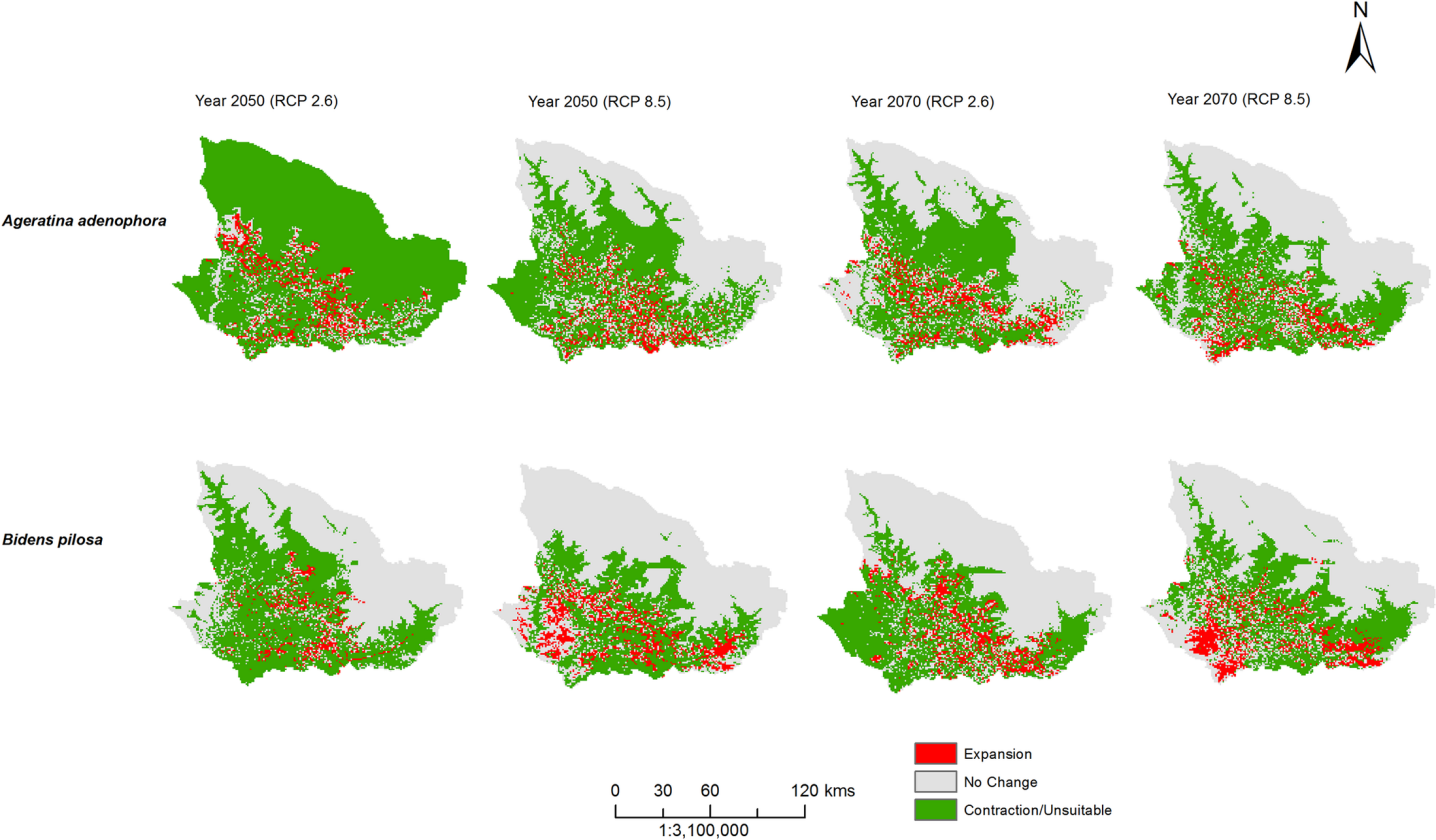
Example of predicted range expansion of *Ageratina adenophora* and *Bidens pilosa* for year 2050 and 2070 at different climatic scenario.

In the beginning of the year 2050, the IAPS will expand towards both upper (1034 m) and lower (246 m) elevation. For instance, *Ageratum conyzoides*, *Erigeron karvinskianus*, *Xanthium strumarium*, *Ageratina adenophora*, and *Bidens pilosa* (Fig 4 and S3 Fig) be speculated to expand upward by 1034 m, 981 m, 800 m, 692 m and 682 m respectively from the current elevation range. Similarly, in the beginning of the year 2070, the IAPS range expand upward by 981 m and downward by 359 m. Hence the elevation range expands from “622 m -2865 m” to “448 m-3547 m”. It is predicted that *A*. *adenophora* and *E*. *karvinskianus* will also expand vertically up to 3547 m and rising 981 m upper elevation than current scenario. While distribution of *Lantana camara* and *Partnenium hysterophorus* exhibited a different trend in the future and a significant decrease in their upper elevation range by 598 m and 486 m respectively in the year 2070. Under future climatic conditions, highest range expansion is predicted in the distribution of *E*. *karvinskianus*, *P*. *hysterophorus*, and *L*. *camara*.

At present *E*. *karvinskianus* is widely distributed and concentrated in central and southeastern parts of the landscape at an elevation range of 677 m to 2566 m with secondary scrub as the dominant vegetation. Under future prediction, it is expected that its distribution will expand in the southern belt, SE of Pithoragarh, SE and SW of Darchula, NE and SE of Baitadi and south of Bajhang. In the future, the distributional range will expand upward by 981m and downward by 200 m. Hence, the distributional elevation range of *E*. *karvinskianus* will be from 477 m to 3547 m. Currently, *L*. *camara* is distributed between 829 m to 1975 m in the central west and SE region of the landscape (along with the border of West Pithoragarh, and NW of Baitadi and South of Darchula districts) in subtropical needle-leaved forest. In future, *L*. *camara* will expand downward by 359 m. The current distribution of *P*. *hysterophorus* is dominant in the central eastern of Pithoragarh, SW of Darchula, patches in south of Baitadi and Bajhang in an elevation range from 686 m to 2050 m predominantly in subtropical needle-leaved forest. In the year 2050 its range will expand by 23 m up and 130 m down compare to present distribution. However, in year 2070, the distributional elevation range expand towards lower elevation up to 238 m down but contracts at an upper elevation by 486 m i.e. the distribution of *P*. *hysterophorus* in year 2070 will be between 448 m to 1564 m. In future, there will be slight expansion in East of Pithoragarh, NE of Baitadi, SE and SW of Bajhang.

## Discussion and conclusions

Research attempt to model the distribution range of 11 IAPS and identify crucial zones for ecosystem management to regulate degradation in part of Western Himalaya. Most of the studies on predicting the distribution of invasive species have focused on one or two species [37, 60–61] and only a few studies have focused on future prediction of more than two species [62–63]. Predicting the future distribution of invasive plants provides insights on their spread under future climate conditions, which in turn can provide the vegetation types prone to degradation because of the invasion. It is essential to identify future invaders and take initial steps in prevention which is the cost-effective means to minimize the spread and impacts of IAPS to new areas.

By year 2050, substantial shifts in bioclimatic conditions is also anticipated throughout the KSL area [14]. Bio-climatic variables like minimum temperature of coldest month, mean temperature of driest quarter and mean diurnal range play major role and contribute more to the invasion by 11 IAPS in the present study. Mostly, the expansion of the invasive species might encroach the natural ecosystems of secondary scrub and subtropical needle-leaved forest. The model predicted that *A*. *adenophora*, *A*. *conyzoides*, *B*. *pilosa*, *E*. *karvinskianus*, and *X*. *strumarium* will significantly expand both vertically and horizontally under all future climate scenarios and invade secondary scrub and subtropical needle-leaved forest as a potential suitable habitat. Compared to the five IAPS mentioned above, *Senna tora* slightly increased in lower elevation (by 6 m) in by year 2050, however, it significantly increased in upper elevation in both year 2050 and 2070. *L*. *camara*, one of the world’s 100 worst invasive species [64] and a problematic invasive species in forest and shrub lands [43] remarkably expanded downward to 359 m while contracting in upper elevation by 474 to 598 m in year 2050 and 2070 respectively in our study area.

*P*. *hysterophorus*, one of the most troublesome weed in the region and one of the worst weed in India [65] has also significantly expanded in Nepal in last 20 years [42, 66] and is rapidly invading in the study area as well [44]. However, in our predicted model for year 2070, *P*. *hysterophorus* upper distribution contracts by 486m but expansion takes place at lower elevation by 238 m. Hence in 2070, the elevation range of *P*. *hysterophorus* will be from 686–2050 m to 448–1564 m and mostly it is invading Subtropical needle-leaved forest.

Our models have predicted present distribution and range expansion under future climate change, which might pose a threat to the native ecosystems in KSL. Assessment of the threats posed by the combined effects of invasive species and climate change [67] is crucial for conservation and management of the biodiversity of KSL. Knowledge and information about the geographical distribution of these species is vital before applying any control measures [68–69].

Mountain ecosystems are still relatively uninvaded by IAPS than lowland ecosystem but the process may be accelerated due to climate change and anthropogenic disturbance. Thus better investigation and planning is needed for early detection and tracking of these species habitat suitability so that appropriate actions can be taken in time to prevent further invasions [8, 10, 70–71].

Effective management of invasive species requires an integrated approach, which includes mechanical, chemical and biological control techniques [72]. For example, invasive species can be easily controlled in the initial stages of establishment when the small satellite populations can be physically removed to stop its further spread [73]. Uprooting the plant before flowering (to control spread by seeds), flooding for short periods of time (*Ageratum conyzoides*), shading by intact canopies (*Lantana camara*) and using herbicides are some of the techniques used to control invasive plant species. Use of biological control agent is one of the tool for management of invasive species. Biological control agents like stem gall fly *Procecidochares utilis* for *Ageratina adenophora* [74] and leaf feeding beetle *Zygogramma bicolorata* for *Parthenium hysterophorus* [75–76] have been used in the management of invasive species. Moreover, for effective management of invasive species, existing biological control strategies should be complemented with suppressive plants [77] and through re-vegetation of degraded sites with competitive native forage grasses [78]. In addition, there is an urgent need to strengthen capacity of scientific community, local community and other stakeholders to control IAPS through identification, prevention, and early detection. Finding alternative use of invasive species by local communities is one of the strategies in managing the invasive species [79]. For example, *L*. *camara* is used to make small scale furniture, bio-briquettes, biochar, farm hedges, fuelwood and as a green manure [80–82]. *A*. *adenophora* has been used as animal bedding, composting, extracting essential oils and odors and have been used as contact poisons or repelling agent’s against herbivore pests in parts of China [43,78]. Invasive plant biomass has been also used for the production of biochar [81–82].

Considering the overall modeling scenario, our results demonstrate that the distribution of IAPS in KSL will expand under future climate change and might be a potential threat to the native vegetation. In the future, the distribution range of the species will expand both vertically and horizontally towards South East and North East of KSL. Our model predicted and identified probable areas and types of vegetation invasion. Most of the range expansion of the IAPS will be in the two natural ecosystems viz., secondary scrub and subtropical needle-leaved forest, this will put these natural ecosystems under threat of resource scarcity. Enhancement of our knowledge and understanding about interaction between native species and invasive species considering climate change is imperative for effective conservation and management ecosystems in the Himalaya.

## Supporting information

S1 Fig Relative importance of environmental variables based on jackknife results. (PDF)

S2 Fig Sensitivity & specificity graph. (PDF)

S3 Fig Predicted range expansion and contraction of IAPS for the year 2050 & 2070 at different climatic scenario. (PDF)

S1 Table Significant variable for predicting invasive species distribution. (DOCX)
