# Supplementary material for: Understanding the dynamics in distribution of invasive alien plant species under predicted climate change in Western Himalaya
Source: PLoS One. 2018 Apr 17;13(4):e0195752. doi: 10.1371/journal.pone.0195752 (PMC5903596; doi:10.1371/journal.pone.0195752)
Supplement: S2 Fig — (PDF) [file pone.0195752.s002.pdf]

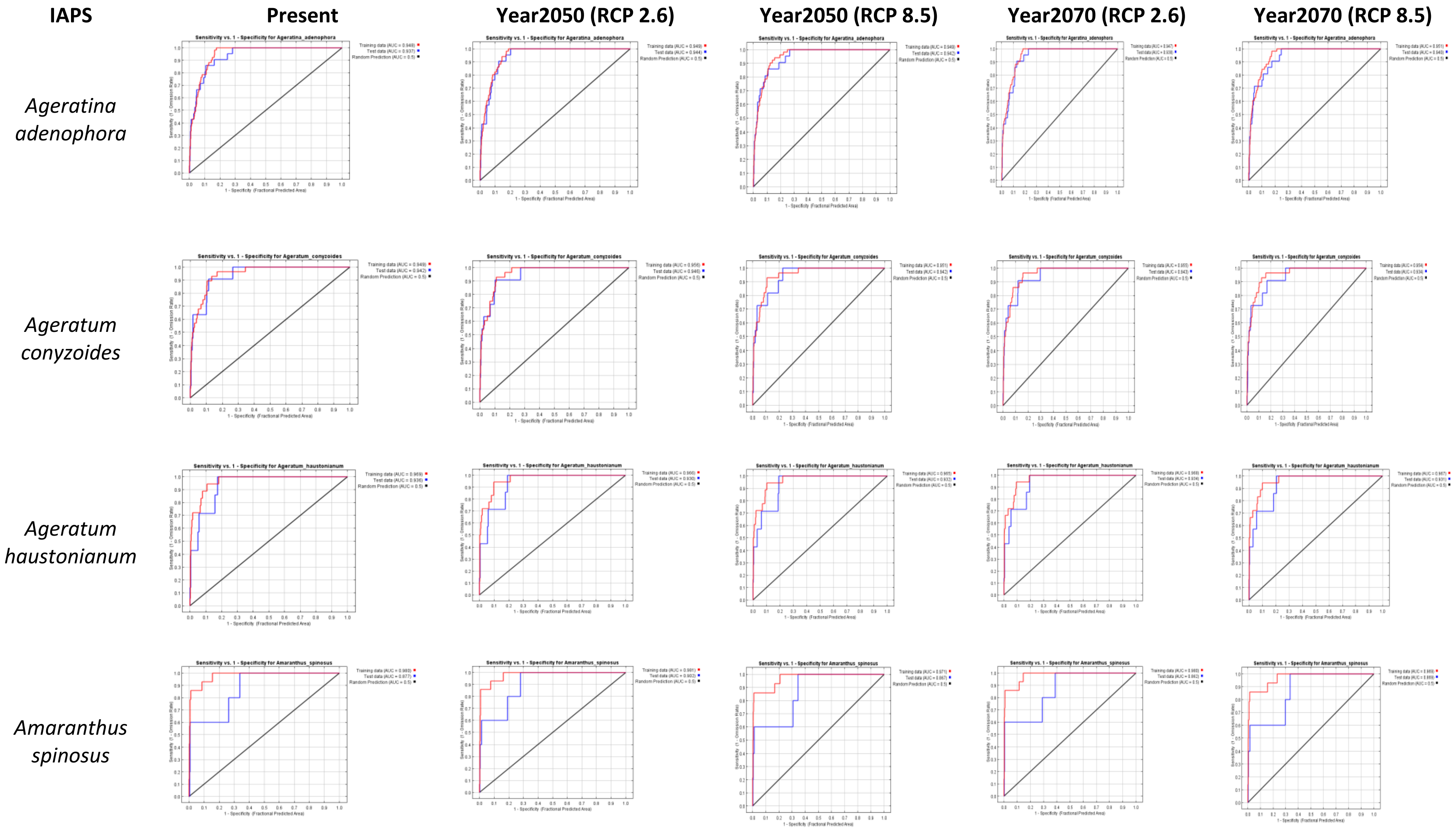
IAPS
Present
Year2050 (RCP 2.6)
Year2050 (RCP 8.5)
Year2070 (RCP 2.6)
Year2070 (RCP 8.5)
Ageratina adenophora
Ageratum conyzoides
Ageratum haustonianum
Amaranthus spinosus

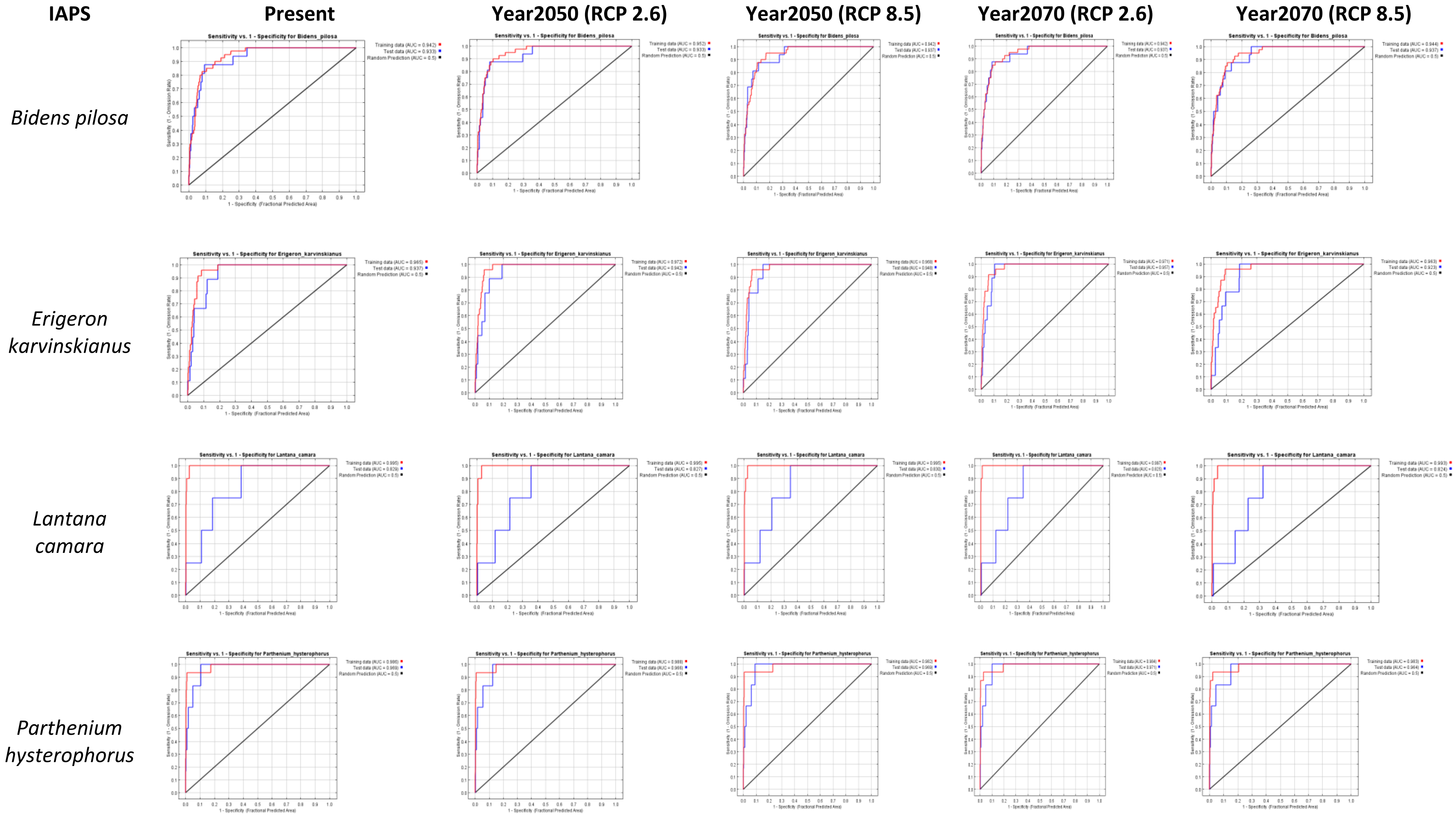
IAPS
Present
Year2050 (RCP 2.6)
Year2050 (RCP 8.5)
Year2070 (RCP 2.6)
Year2070 (RCP 8.5)
Bidens pilosa
Erigeron karvinskianus
Lantana camara
Parthenium hysterophorus
Sensitivity vs. 1 - Specificity for Bidens_pilosa
Sensitivity vs. 1 - Specificity for Erigeron_karvinskianus
Sensitivity vs. 1 - Specificity for Lantana_camara
Sensitivity vs. 1 - Specificity for Parthenium_hysterophorus
1 - Specificity (Fractional Predicted Area)
Sensitivity (1 - Omission Rate)

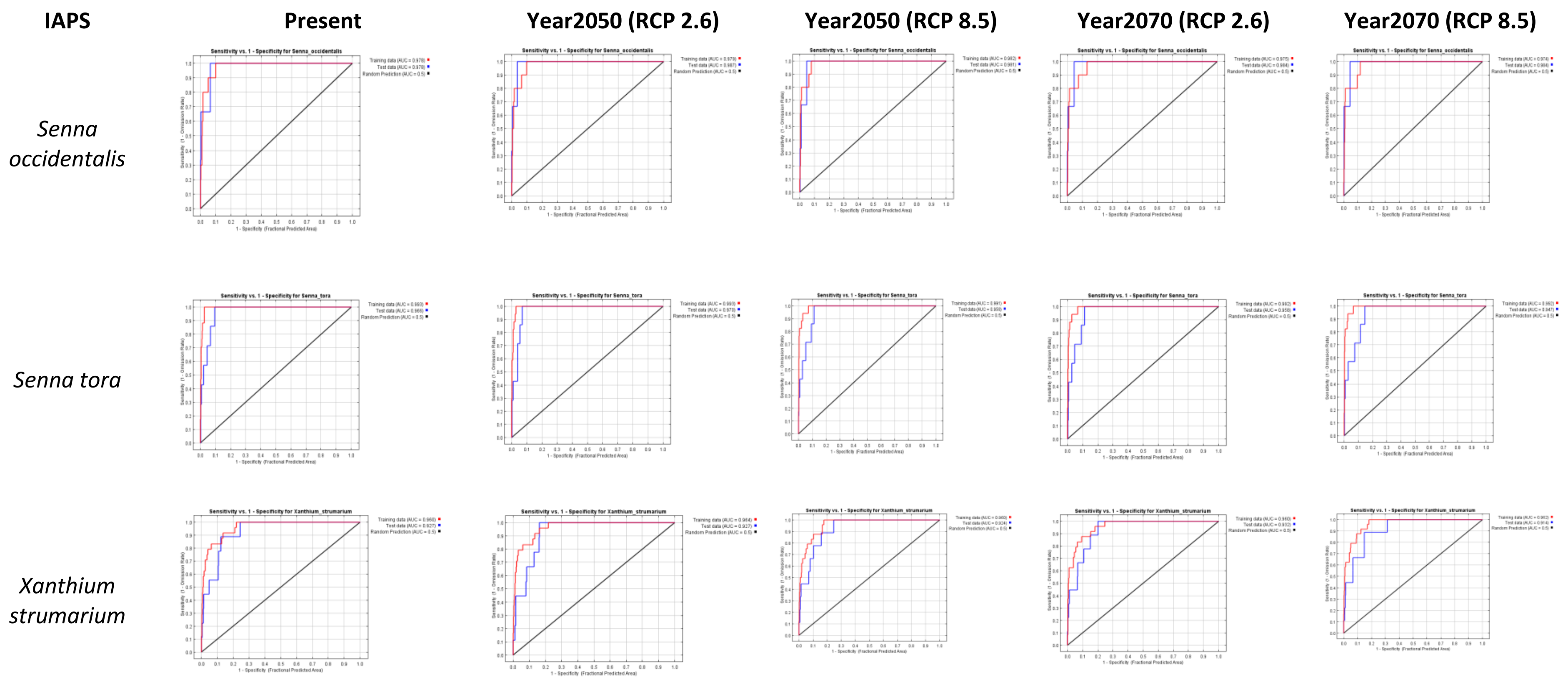
IAPS
Present
Year2050 (RCP 2.6)
Year2050 (RCP 8.5)
Year2070 (RCP 2.6)
Year2070 (RCP 8.5)
Senna occidentalis
Senna tora
Xanthium strumarium
Sensitivity vs. 1 - Specificity for Senna_occidentalis
Sensitivity vs. 1 - Specificity for Senna_tora
Sensitivity vs. 1 - Specificity for Xanthium_strumarium
1 - Specificity (Fractional Predicted Area)
Sensitivity (1 - Omission Rate)
