# Supplementary material for: Understanding the dynamics in distribution of invasive alien plant species under predicted climate change in Western Himalaya
Source: PLoS One. 2018 Apr 17;13(4):e0195752. doi: 10.1371/journal.pone.0195752 (PMC5903596; doi:10.1371/journal.pone.0195752)
Supplement: S3 Fig — (PDF) [file pone.0195752.s003.pdf]

**S3 Figure: Predicted range expansion and contraction of IAPS for the year 2050 & 2070 at different climatic scenario**

**Species Name** | **Year 2050 ( R.C.P. 2.6)** | **Year 2050 (R.C.P. 8.5)** | **Year 2070(R.C.P. 2.6)** | **Year 2070 (R.C.P.8.5)**

*Ageratina adenophora* L.

*Ageratum conyzoides* L.

*Ageratum houstonianum* Mill.

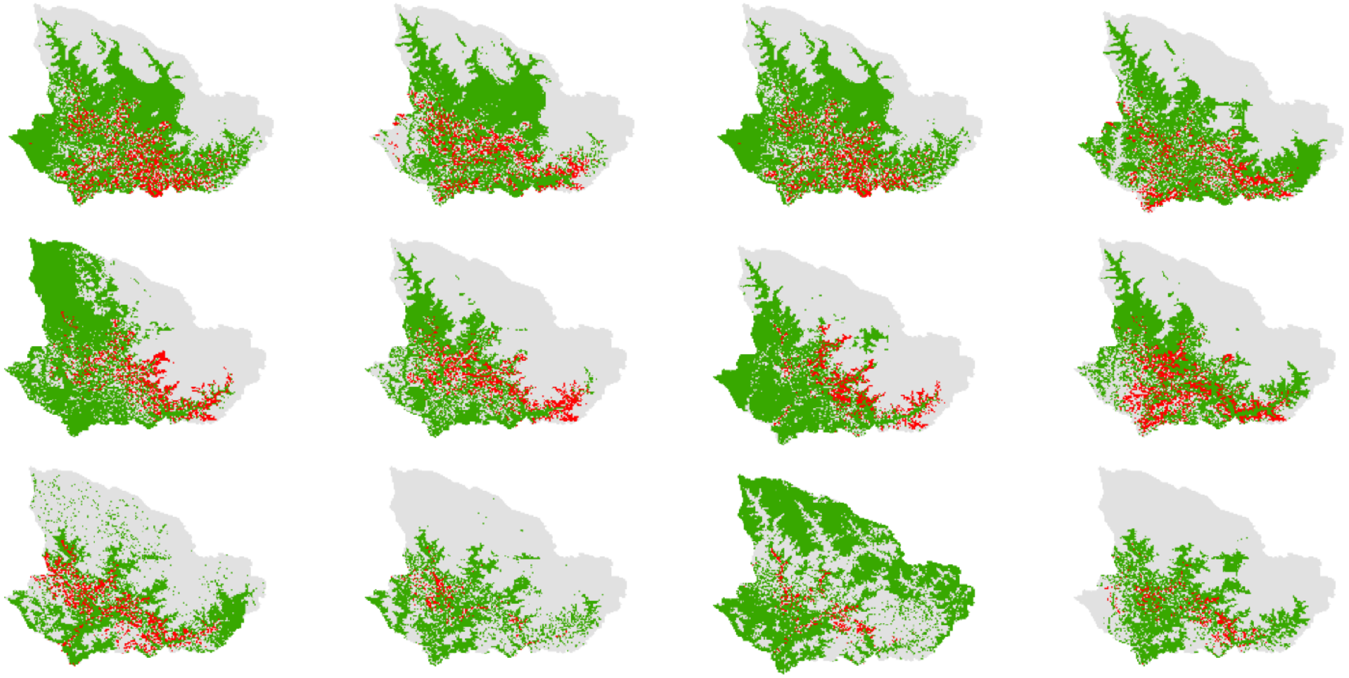

Expansion

No Change

Contraction/Unsuitable

| Species Name | Year 2050 ( R.C.P. 2.6) | Year 2050 (R.C.P. 8.5) | Year 2070 (R.C.P. 2.6) | Year 2070 (R.C.P.8.5) |
|---|---|---|---|---|
| *Amaranthus spinosus L.* | 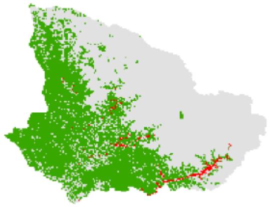 | 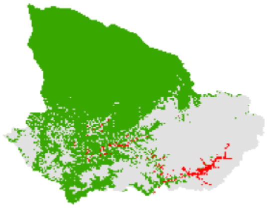 | 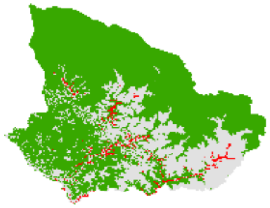 | 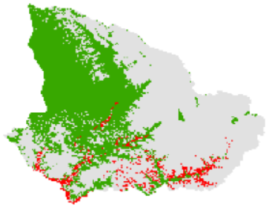 |
| *Bidens pilosa* L. | 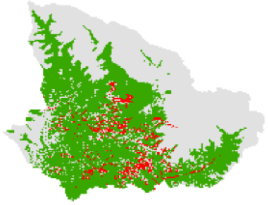 | 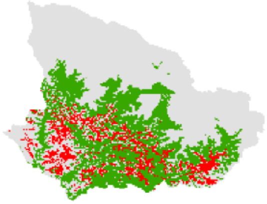 | 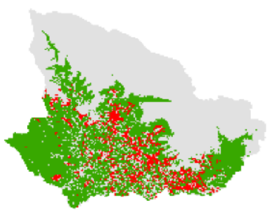 | 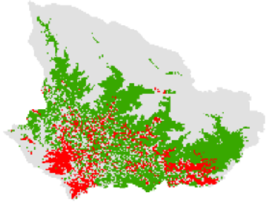 |
| *Erigeron karvinskianus* DC. | 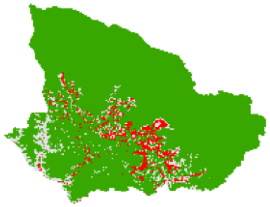 | 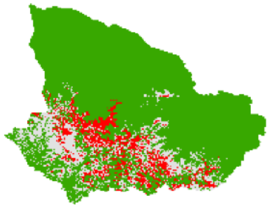 | 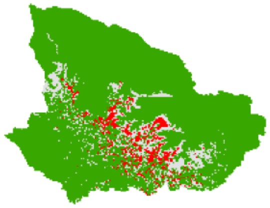 | 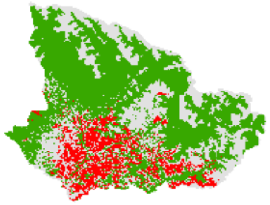 |
| *Lantana camara* L. | 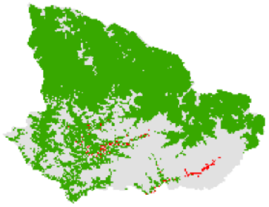 | 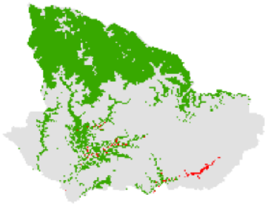 | 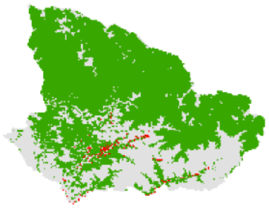 | 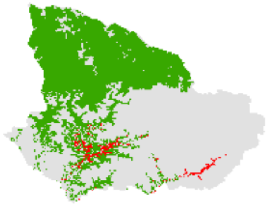 |

| **Species Name** | **Year 2050 ( R.C.P. 2.6)** | **Year 2050 (R.C.P. 8.5)** | **Year 2070 (R.C.P. 2.6)** | **Year 2070 (R.C.P.8.5)** |
| --- | --- | --- | --- | --- |
| *Parthenium hysterophorus* L. | 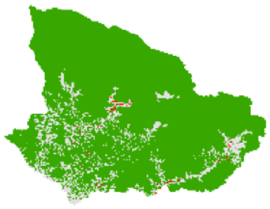 | 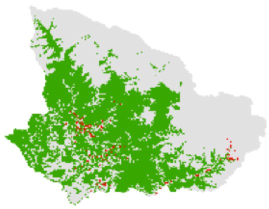 | 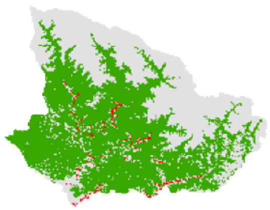 | 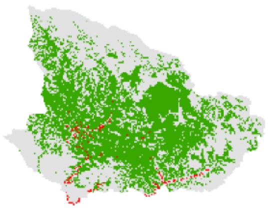 |
| *Senna occidentalis* (L.) Link | 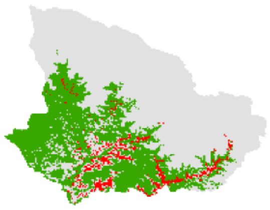 | 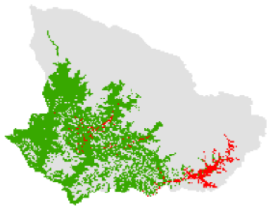 | 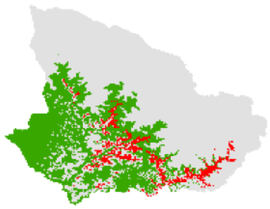 | 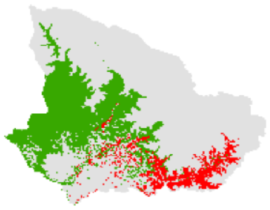 |
| *Senna tora* (L.) Roxb | 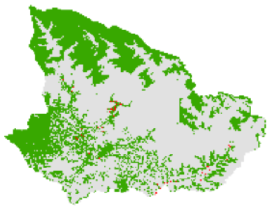 | 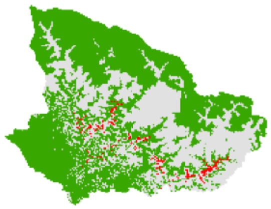 | 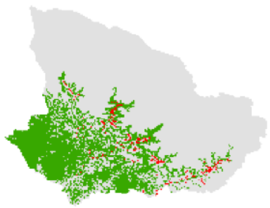 | 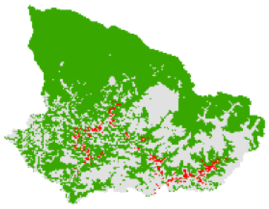 |
| *Xanthium strumarium* L. | 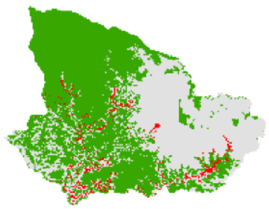 | 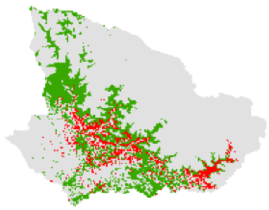 | 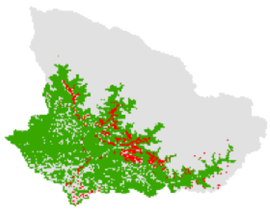 | 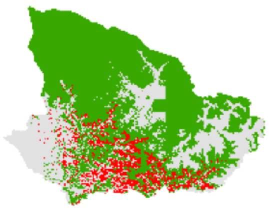 |
