# Supplementary material for: Understanding the dynamics in distribution of invasive alien plant species under predicted climate change in Western Himalaya
Source: PLoS One. 2018 Apr 17;13(4):e0195752. doi: 10.1371/journal.pone.0195752 (PMC5903596; doi:10.1371/journal.pone.0195752)
Supplement: S1 Table — (DOCX) [file pone.0195752.s004.docx]

S1 Table: Significant variable for predicting invasive species distribution

| S.N. | Species | Sample/Training Number | Present Year | | | Year 2050 (CCSM4) | | | | | | Year 2070 (CCSM4) | | | | | |
| --- | --- | --- | --- | --- | --- | --- | --- | --- | --- | --- | --- | --- | --- | --- | --- | --- | --- |
|  |  |  | AUC value | | Significant Variable (Jacknife) | RCP 2.6 | | | RCP 8.5 | | | RCP 2.6 | | | RCP 8.5 | | |
|  |  |  |  |  |  | AUC value | | Significant Variable (Jacknife) | AUC value | | Significant Variable (Jacknife) | AUC value | | Significant Variable (Jacknife) | AUC value | | Significant Variable (Jacknife) |
|  |  |  | Training Data | Test Data |  | Training Data | Test Data |  | Training Data | Test Data |  | Training Data | Test Data |  | Training Data | Test Data |  |
| 1 | *Ageratina adenophora* | 130 | 0.94 | 0.93 | bio 9 | 0.94 | 0.94 | bio 9 | 0.94 | 0.94 | bio 6 | 0.94 | 0.93 | bio 9 | 0.95 | 0.9 | bio 6 |
| 2 | *Ageratum conyzoides* | *69* | 0.94 | 0.94 | bio 2 | 0.95 | 0.94 | bio 2 | 0.95 | 0.94 | bio 2 | 0.95 | 0.94 | bio 9 | 0.95 | 0.93 | bio 2 |
| 3 | *Ageratum houstonianum* | 35 | 0.96 | 0.93 | bio 9 | 0.96 | 0.93 | bio 9 | 0.96 | 0.93 | bio 6 | 0.96 | 0.93 | bio 9 | 0.96 | 0.93 | bio 6 |
| 4 | *Amaranthus spinosus* | *19* | 0.98 | 0.87 | bio 2 | 0.98 | 0.90 | bio 2 | 0.95 | 0.94 | bio 2 | 0.98 | 0.86 | bio 2 | 0.96 | 0.86 | bio 2 |
| 5 | *Bidens pilosa* | *101* | 0.94 | 0.93 | bio 9 | 0.95 | 0.93 | bio 2 | 0.97 | 0.86 | bio 2 | 0.94 | 0.93 | bio 9 | 0.94 | 0.93 | bio 6 |
| 6 | *Erigeron karvinskianus* | *96* | 0.96 | 0.93 | bio 9 | 0.97 | 0.94 | bio 19 | 0.94 | 0.93 | bio 6 | 0.97 | 0.95 | bio 9 | 0.96 | 0.92 | bio 19 |
| 7 | *Lantana camara* | *20* | 0.99 | 0.82 | bio 8 | 0.99 | 0.82 | bio 8 | 0.99 | 0.83 | bio 8 | 0.99 | 0.82 | bio 8 | 0.99 | 0.82 | bio 8 |
| 8 | *Parthenium hysterophorus* | *25* | 0.98 | 0.96 | bio 2 | 0.98 | 0.96 | bio 2 | 0.98 | 0.96 | bio 2 | 0.98 | 0.97 | bio 2 | 0.98 | 0.96 | bio 2 |
| 9 | *Senna occidentalis* | *15* | 0.97 | 0.97 | bio 8 | 0.97 | 0.98 | bio 2 | 0.98 | 0.98 | bio 6 | 0.97 | 0.98 | bio 2 | 0.97 | 0.98 | bio 6 |
| 10 | *Senna tora* | *28* | 0.99 | 0.96 | bio 9 | 0.99 | 0.97 | bio 9 | 0.99 | 0.95 | Elevation | 0.99 | 0.95 | Elevation | 0.99 | 0.94 | bio 8 & elevation |
| 11 | *Xanthium strumarium* | 40 | 0.96 | 0.92 | bio 9 | 0.96 | 0.92 | bio 5 | 0.96 | 0.92 | bio 6 | 0.96 | 0.93 | bio 9 | 0.96 | 0.91 | bio 6 |
